# The Impact of Racial and Ethnic Background on Overall Survival in Endometrial Cancer Patients Receiving Immunotherapy as Front-Line Treatment

**DOI:** 10.3390/cancers18182914

**Published:** 2026-09-09

**Authors:** Keizra Mecklai, Iman Osman, Esther Adler, Sarah S. Lee

**Affiliations:** 1 Department of Obstetrics and Gynecology, Grossman School of Medicine, New York University, 159 E 53rd Street, 2nd Floor, New York, NY 10022, USA; keizra.mecklai@nyulangone.org; 2 Department of Dermatology, Grossman School of Medicine, New York University, 522 First Avenue, 4th Floor, New York, NY 10016, USA; 3 Department of Pathology, Grossman School of Medicine, New York University, 550 First Avenue, Suite TH461, New York, NY 10016, USA; esther.adler@nyulangone.org

**Keywords:** endometrial cancer, race/ethnicity, immunotherapy, immune checkpoint inhibitors, disparities, overall survival

## Abstract

**Background/Objective:** For decades, non-Hispanic Black and Hispanic patients have been reported to have worse outcomes in endometrial cancer compared to non-Hispanic White patients. More recently, the introduction of immunotherapy has improved outcomes for patients with advanced and recurrent endometrial cancer. In this study, we examined the impact of race and ethnicity on overall survival in patients with endometrial cancer who received immunotherapy. **Methods:** Patients with endometrial cancer who received immunotherapy between 2019 and 2022 were identified in the National Cancer Database. Demographic, socioeconomic, and clinicopathological characteristics were evaluated. The association between race/ethnicity and overall survival was examined using Kaplan–Meier and multivariable Cox regression. **Results:** We identified 4322 patients (68% non-Hispanic White, 21% non-Hispanic Black, 7% Hispanic, and 4% Asian). Non-Hispanic Black patients had similar overall survival rates compared to non-Hispanic White patients (aHR 1.03, 95% CI 0.86–1.23), despite having the highest frequency of serous histology, an aggressive subtype (51% vs. 36%, *p* < 0.001). Hispanic patients, who were younger (*p* < 0.001) and had a higher frequency of Medicaid insurance compared to the overall cohort (*p* < 0.001), had worse survival compared to non-Hispanic White patients (aHR 1.37, 95% CI 1.05–1.80) in a multivariate model controlling for differences in demographic, socioeconomic, and clinical variables. **Conclusions:** For endometrial cancer patients receiving immunotherapy, there were no differences in survival between non-Hispanic White and non-Hispanic Black patients. However, Hispanic patients continue to experience significantly worse outcomes that cannot be explained only by disparity in access to novel treatments. More studies are warranted to better identify the causes driving the differences to improve outcomes for all patients.

## Simple Summary

Non-Hispanic Black and Hispanic patients continually have worse outcomes in endometrial cancer than non-Hispanic White patients. Immunotherapy has improved outcomes for patients with endometrial cancer, but the impact on racial disparities is unknown. In this study, we examined the impact of race and ethnicity on overall survival among patients with endometrial cancer who were treated with immunotherapy in the United States. Findings suggest that while immunotherapy treatment reduces the disparity in overall survival between non-Hispanic Black and non-Hispanic White patients, Hispanic patients continue to experience significantly worse outcomes. This highlights the importance of ensuring equitable access to early diagnosis, molecular testing, and emerging therapies as well as the need for additional research to identify the drivers of outcome disparities in endometrial cancer.

## 1. Introduction

In 2026, 68,270 women are projected to be diagnosed with endometrial cancer, and 14,450 women are expected to die from the disease [1]. Rates of endometrial cancer have increased by approximately 1% per year since the mid-2000s, and mortality has increased 1.5% per year due to the rise in aggressive histologic subtypes, particularly in Hispanic and non-Hispanic Black patients [1,2,3]. Hispanic and non-Hispanic Black patients are more likely to be diagnosed at advanced stages, have a greater proportion of aggressive histology, are less likely to receive guideline-concordant care, and experience more delays in care than patients in other racial or ethnic groups [4,5,6,7,8]. Prior studies examining disparities in endometrial cancer have largely focused on inequities in access to care and receipt of guideline-concordant treatment.

Immune checkpoint inhibitors have revolutionized the treatment landscape for advanced or recurrent endometrial cancer, especially for microsatellite instability high (MSI-H- see abbreviation list) or mismatch repair deficient (dMMR) tumors. While single-agent immune checkpoint inhibitors have been approved for MSI-H/dMMR recurrent disease since 2022, subsequent trials have established the efficacy of immune checkpoint inhibitors and chemotherapy as first-line treatment for advanced endometrial cancer [9,10,11,12,13]. Yet, the impact of race and ethnicity on endometrial cancer outcomes in the new immunotherapy era is unknown.

The objective of this study was to characterize the demographic, socioeconomic, and clinical factors of patients with endometrial cancer who have received immunotherapy in the last four years of available data and to evaluate the impact of race and ethnicity on overall survival.

## 2. Materials and Methods

Adult patients with endometrial cancer diagnosed between January 2019 and December 2022 registered in the National Cancer Database (NCDB) and treated with immunotherapy were included. The NCDB is a publicly available, United States (US)-based outcomes database that contains de-identified oncologic and sociodemographic patient data from over 1500 Commission on Cancer-accredited centers [14]. The NCDB captures facility-level data on approximately 72% of all newly diagnosed cancer cases in the United States [15]. Data from 2019 onward were included, as phase III clinical trials evaluating the efficacy of immune checkpoint inhibitors in endometrial cancer began recruiting that year [9,11]. At the time of analysis, 2022 was the most recent year for which data abstraction was available.

NCDB reports race (variable: RACE) and ethnicity (variable: SPANISH_HISPANIC_ORIGIN) as Spanish/Hispanic origin. We reported race and ethnicity groups per the US Office of Management and Budget categories: American Indian or Alaska Native (American Indian, Aleutian, Other), Asian (Chinese, Japanese, Filipino, Korean, Vietnamese, Laotian, Hmong, Kampuchean, Thai, Asian Indian or Pakistani, Other Asian), non-Hispanic Black or African American, Hispanic or Latino Native Hawaiian or Pacific Islander (Hawaiian, Micronesian, Chamorran, Guamanian, Polynesian, Tahitian, Samoan, Tongan, Melanesian, Fiji Islander, New Guinean, and Pacific Islander), non-Hispanic White, and Other race/ethnicity (other or unknown race). Patients identified as Non-Hispanic White, non-Hispanic Black, Asian, and Hispanic were included. Patients identified as American Indian and Native Hawaiian/Pacific Islander were excluded from the analysis per the NCDB suppression threshold and data reporting policy to protect patient privacy, given the small cell counts. Area-level socioeconomic measures were derived from the patient’s zip code of residence using data from the 2016 US census. Education attainment was measured as the proportion of adults within the patient’s zip code who graduated from high school and was categorized into quartiles among all US zip codes [16,17,18,19]. Median household income was similarly estimated at the zip code level and categorized into quartiles.

Immunotherapy use was identified using the variable ‘Immunotherapy (RX_SUMM_IMMUNOTHERAPY)’ as described in the NCDB data dictionary, which included any immunotherapy prescribed as first-line treatment at the reporting or any other facility [15]. The NCDB only reports front-line treatment and does not report recurrence, subsequent treatment lines, and progression-free survival. Therefore, immunotherapy or other treatments in the recurrent setting were not included. All patients, regardless of whether they were enrolled in a clinical trial, were included. Clinical trial enrollment was defined using the variable ‘Other Treatment (RX_SUMM_OTHER)’ in the data dictionary, in accordance with prior methodology established by Khadraoui et al. [15,20]. Reasons for immunotherapy use and the nuances of clinician and patient decision-making were not available.

Patients with 2009 International Federation of Gynecology and Obstetrics (FIGO) stage I through IV endometrial cancer of endometrioid, serous, carcinosarcoma, and other histologies (e.g., clear cell, mucinous, dedifferentiated, carcinoma not otherwise specified) were included in the analysis, while patients with endometrial precancer or sarcoma were excluded. Patients with missing or unknown survival outcomes were excluded from the overall survival analysis.

The primary outcome was the association between overall survival and race/ethnicity for patients with endometrial cancer receiving immunotherapy. Overall survival was first analyzed by comparing non-Hispanic White patients with Asian, Hispanic, and non-Hispanic Black patients, adjusting for demographic, socioeconomic, and clinical factors. We secondarily examined the demographic, socioeconomic, and clinical characteristics of patients receiving immunotherapy by race/ethnicity.

Data were analyzed using Stata version 17 (College Station, TX, USA). Chi-square tests were used to analyze differences in proportions of patients receiving immunotherapy by demographic and clinical factors. Kaplan–Meier curves were used to estimate overall survival by race/ethnicity. Multivariable Cox proportional hazards regression was used to assess the association between race and ethnicity and overall survival, while adjusting for demographic, socioeconomic, and clinical characteristics. Covariates included age, insurance status, area-level median household income and educational attainment, comorbidity, distance from treatment facility, facility type, geographic region, stage, and histology. The covariates were selected a priori based on clinical relevance and their potential association with treatment and survival. Adjusted hazard ratios and 95% confidence intervals were reported. *p* value less than 0.05 was considered statistically significant. Sensitivity analyses were performed for patients with advanced-stage disease and for those who received chemotherapy plus immunotherapy as front-line treatment.

In accordance with the NCDB data use agreement, cells with fewer than 10 patients were suppressed to protect patient confidentiality. For variables with missing or unknown categories containing fewer than 10 patients, these categories were excluded from descriptive analyses, and percentages were calculated among patients with non-missing data. This study was deemed exempt from the Institutional Review Board review. As the data are publicly available and de-identified, a waiver of informed consent was not required. Data are available upon reasonable request and are subject to NCDB restrictions.

## 3. Results

### 3.1. Patient Cohort

We identified 191,708 patients diagnosed with endometrial cancer in the NCDB between 2019 and 2022. Of those, 4467 patients (2%) received immunotherapy, with increasing immunotherapy use noted over time (2019: 705 [1%], 2020: 1017 [2%], 2021: 1239 [2%], 2022: 1506 [3%]). The median time from diagnosis to immunotherapy administration was 90 days (IQR: 55–163 days).

Immunotherapy use differed by race/ethnicity, with 1% of American Indian, Alaskan Native patients receiving immunotherapy compared to 3% of Asian patients, 2% of Hispanic patients, 3% of Native Hawaiian and Pacific Islander patients, 4% of non-Hispanic Black patients, 2% of non-Hispanic White patients, and 2% of Other patients (*p* < 0.001). Due to small sample sizes in the American Indian, Alaskan Native, Native Hawaiian and Pacific Islander, and Other race/ethnicity groups, these groups were not included in further analysis per NCDB guidelines on data suppression for small population sizes to protect patient privacy. The final cohort for analysis by race/ethnicity included 4322 patients who received immunotherapy; the majority of whom were non-Hispanic White (68%, *n* = 2936), followed by non-Hispanic Black (21%, *n* = 912), Hispanic (7%, *n* = 292), and Asian (4%, *n* = 182).

### 3.2. Demographic and Socioeconomic Characteristics of Patients Receiving Immunotherapy

Table 1 describes the demographic and socioeconomic characteristics of patients receiving immunotherapy by race and ethnicity. While the distribution of age at diagnosis was similar among Asian, non-Hispanic Black, and non-Hispanic White patients, Hispanic patients were younger at diagnosis, with 20% diagnosed at 18–49 years compared to the overall cohort (6%, *p* < 0.001). Hispanic patients were more likely to be insured by Medicaid (20%) than Asian (12%), non-Hispanic Black (12%), and non-Hispanic White (6%) patients (*p* < 0.001). More than half of Asian (63%) and non-Hispanic White (54%) patients lived in neighborhoods with higher educational attainment, as defined by the proportion living in zip codes with above-median high school graduation rates, compared to less than a third of Hispanic (25%) and non-Hispanic Black (29%, *p* < 0.001). Asian patients were more likely to reside in neighborhoods with income above the US median (78%), and Hispanic and non-Hispanic Black patients were less likely (43% and 37%, *p* < 0.001). Non-Hispanic White (17%) and Hispanic (10%) patients were more likely to live more than 50 miles from the treatment facility than non-Hispanic Black (7%) and Asian patients (6%, *p* < 0.001). Hispanic and non-Hispanic Black patients were more likely to have received care in an academic/research facility (Hispanic: 58%, non-Hispanic Black: 53%, non-Hispanic White: 42%, Asian: 40%, *p* < 0.001).

### 3.3. Clinical Characteristics of Patients Receiving Immunotherapy

The clinical characteristics of patients who received immunotherapy, analyzed by race and ethnicity, are described in Table 2. Most (89%) patients had advanced stage disease at diagnosis. Half of the patients had aggressive histology (serous, 40%; carcinosarcoma, 7%). Endometrioid histology was seen in a minority of the cohort, with 29% (*n* = 1258). Notably, half of non-Hispanic Black patients had serous histology (51%), compared to a third of Asian, Hispanic, and non-Hispanic White patients (*p* < 0.001). Most patients (93%) received both chemotherapy and immunotherapy as part of their initial treatment course. Non-Hispanic Black (96%) and Asian (94%) patients were more likely to receive concurrent chemotherapy with immunotherapy compared to non-Hispanic White (91%) and Hispanic (92%) patients in this cohort (*p* < 0.001). Only 1% of the patients indicated clinical trial enrollment. Non-Hispanic White patients were more likely to be enrolled in a clinical trial than patients of all other races/ethnicities (2% vs. 1%, *p* = 0.015).

### 3.4. Overall Survival Analysis of Patients Receiving Immunotherapy

**Figure 1:**
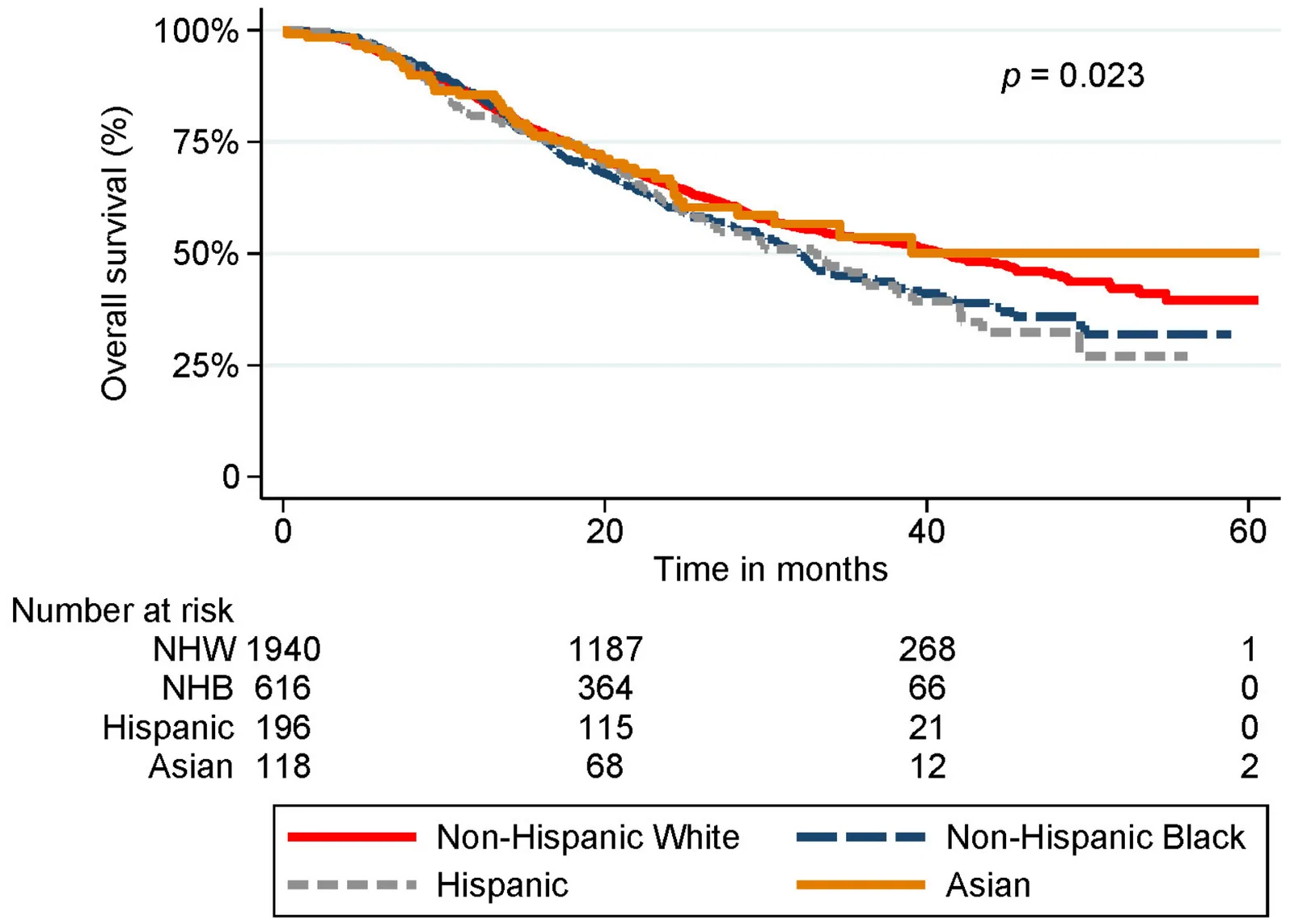
Kaplan–Meier plot of overall survival by racial/ethnic groups (Non-Hispanic White vs. Non-Hispanic Black vs. Hispanic vs. Asian).

The overall survival by disaggregated racial and ethnic groups was as follows: Asian: not reached, (95% CI 25—not reached); non-Hispanic White: 41 months (95% CI 38–45); Hispanic: 33 months (95% CI 26–39); and non-Hispanic Black: 32 months (95% CI 30–34) (*p* = 0.023) (Figure 1). However, when controlling for demographic, socioeconomic, and clinical factors, in this cohort of patients receiving immunotherapy, Hispanic patients had persistently worse survival than non-Hispanic White patients (aHR 1.37, 95% CI 1.05–1.80). In contrast, the survival difference between non-Hispanic Black patients and non-Hispanic White patients was no longer present with immunotherapy receipt when controlling for demographic, socioeconomic, and clinical factors (aHR 1.03, 95% CI 0.86–1.23). There were no differences in survival between Asian and non-Hispanic White patients (aHR 1.12, 95% CI 0.78–1.61).

### 3.5. Factors Associated with Overall Survival

Factors associated with overall survival among patients with endometrial cancer who received immunotherapy are described in Table 3. Patients who received care in the Midwest were more likely to die of their disease than those who received care in the Northeast (aHR 1.30, 95% CI 1.07–1.59). Unsurprisingly, patients with advanced disease (stage III–IV) had worse overall survival than patients with early-stage disease (stage I–II) (aHR 3.10, 95% CI 2.27–4.26). Patients with more aggressive histology fared worse than patients with endometrioid histology. There were no associations between insurance status, education, income, Charlson comorbidity score, distance from treatment facility, facility type, or urban density and survival. In a sensitivity analysis of advanced-stage patients and of patients who received concurrent chemotherapy, there were no differences in the primary findings.

## 4. Discussion

In this large, contemporary United States-based cohort, we characterized real-world immunotherapy treatment for endometrial cancer and examined racial and ethnic differences in outcomes. Most patients who received immunotherapy had advanced-stage disease, aggressive histology, were treated at academic medical centers, had higher socioeconomic status, and resided in metropolitan areas. Hispanic patients, who were younger, more likely to be Medicaid-insured, and lived in areas of lower educational attainment, experienced significantly worse survival than non-Hispanic White patients. In contrast, despite having a higher proportion of patients with serous histology, a known aggressive subtype, disparities between non-Hispanic Black and non-Hispanic White patients were attenuated for patients receiving front-line immunotherapy. Asian patients experienced overall survival comparable to that of non-Hispanic White patients.

Our findings suggest that in the immunotherapy era, the benefit of these novel treatments impacts racial and ethnic groups differently. Despite having a higher proportion of patients with serous histology, when receiving immunotherapy, non-Hispanic Black patients with endometrial cancer had survival outcomes in parity with non-Hispanic White patients. Prior studies support these findings. In a post-hoc analysis of the AtTEnd trial comparing the efficacy of atezolizumab and chemotherapy for advanced and recurrent endometrial cancers between Asian and non-Asian patients, Asian patients had improved outcomes with atezolizumab in dMMR tumors, but the benefit was reduced in Asian patients with pMMR tumors [21]. In a NCDB study in patients with non-small cell lung cancer receiving immunotherapy, non-Hispanic Black patients had a 25% lower risk of death compared to non-Hispanic White patients [22]. In our cohort, non-Hispanic Black patients were more likely to be treated at an academic network compared to non-Hispanic White patients. Similar patterns have been demonstrated in patients with melanoma and non-small cell lung cancer, with those treated at academic medical centers and those living in areas of higher income and educational attainment more likely to receive immunotherapy [18,23]. Treatment at low-volume centers has been associated with less guideline-concordant care and worse outcomes for endometrial cancer patients, though hospital volume only mitigated, but did not eliminate, disparities in outcomes for Black patients [24,25]. Though treatment-center volume was not examined in our study, our study suggests that when equitable access to novel treatments can be achieved, non-Hispanic Black patients may experience comparable outcomes to those of non-Hispanic White patients.

In contrast, in our cohort of endometrial cancer patients receiving immunotherapy, Hispanic patients had significantly worse survival compared to non-Hispanic White patients, even when controlling for factors such as age, insurance, treating facility, stage, and histology. Similar patterns have been reported in other cancers. In lung and head and neck cancers, Hispanic patients had lower response rates compared to non-Hispanic White and non-Hispanic Black patients, whereas in renal cell carcinoma, there were no significant differences [26,27]. These data suggest that in the setting of equal access to novel treatments, the impact of immunotherapy may differentially impact racial and ethnic groups.

In our study, Hispanic patients were more likely to be younger and have Medicaid insurance, but experienced worse comparable survival compared to other racial/ethnic groups. These findings need to be taken in the context that Hispanics are not a monolithic group but are genetically diverse populations with varying proportions of Indigenous American, European, and African ancestry [28]. Currently comprising 20% of the US national population with 68 million individuals, the Hispanic population is projected to reach 28% by 2060, and two-thirds of Hispanics speak a language other than English at home, the highest of any racial or ethnic group [29,30]. Patients with limited English proficiency experience challenges accessing and navigating the complexity of the cancer care spectrum, from diagnosis to treatment, resulting in inadequate consent processes and patient education [31]. A single-institution retrospective series exploring outcomes of English-speaking compared to non-English-speaking endometrial cancer patients demonstrated worse recurrence-free and overall survival in non-English-speaking patients [32]. Although language proficiency is unable to be assessed in our study, we hypothesize that additional factors, both structural and biological, warrant further exploration in future studies to ensure that all patients benefit from novel therapies.

Our study has several strengths. First, we used data from the NCDB, which captures >70% of all newly diagnosed cancer cases in the United States each year. All patients in our cohort received immunotherapy; thus, we were able to explore factors associated with outcomes, independent of disparities in access to novel treatments, and to highlight factors associated with overall survival that can inform future targeted investigations. Second, one-third of patients who received immunotherapy were from diverse racial and ethnic backgrounds, representing a more heterogeneous cohort than in most trials assessing immunotherapy for patients with endometrial cancer. Finally, the use of real-world data reflects the realities of integrating new therapies outside a controlled setting, which may be more generalizable at the population level.

This study was limited by the lack of detailed information in the NCDB, including the specific immunotherapy agent, treatment duration, dosing, toxicity, and discontinuation. Given that the NCDB only reports first-line treatment data, the use of immunotherapy in recurrent endometrial cancer, the only FDA-approved indication for immune checkpoint inhibitors during the study period, was not captured, and the differences in guideline-concordant treatment could not be evaluated [12]. As a result, progression-free survival could not be assessed and thus could not be utilized as a predictor of immunotherapy response, a key limitation in this study. The NCDB also does not report disease recurrence, which may be a better predictor of immunotherapy response. The NCDB does not report molecular subtype information for endometrial cancers, specifically MSI-H/dMMR status, which are known biomarkers that influence immunotherapy eligibility and efficacy, which is a central limitation to interpreting the impact of immunotherapy response. To approximate clinical trial enrollment, we used the variable ‘Other Treatment (RX_SUMM_OTHER)’, a methodology previously established by Khadraoui et al. [20]. However, this is a limited variable, as it captures only the presence or absence of clinical trial enrollment, not the nuances of the treatments, trials, or enrollment in subsequent lines of therapy. Education attainment and median household income were measured at the zip code level and therefore characterize patients’ area of residence rather than individual patients and may not accurately reflect an individual’s socioeconomic status. Given the NCDB guidelines on reporting of small cell data to protect patient privacy, the analysis excluded data from American Indian and Alaska Native, Native Hawaiian/Pacific Islander, and Other race/ethnicity groups, which limits generalizability; moreover, the numbers of Hispanic and Asian patients were overall low, limiting the precision estimates of the adjusted hazard ratios. Lastly, this study included a relatively short follow-up period from 2019 to 2022, given the delayed release of NCDB data. Thus, the data may overestimate overall survival. Further studies are needed to validate these findings as data become available during the FDA approval period, with longer-term follow-up.

Finally, whereas race is often a proxy for socioeconomic status, environmental exposures, and structural barriers that influence diagnosis, treatment, and survival, genetic ancestry reflects inherited genomic variation that may capture biological differences. Race and ethnicity should not be interpreted as causal biological factors for cancer outcomes. Genetic ancestry, therefore, may provide a more appropriate framework for exploring potential biological contributors. The contribution of genetic ancestry to immunotherapy response in patients with endometrial cancer is currently unknown. Moreover, while our findings suggest differences in outcomes among those who received immunotherapy across racial and ethnic subgroups, these observations remain hypothesis-generating, given the limitations of self-identified race as a proxy for underlying genomic variation, and warrant further investigation with a prospective, ancestry-enriched cohort, with molecular information and treatment-line data.

## 5. Conclusions

Overall, immunotherapy may attenuate but not eliminate disparities in overall survival in all patients with advanced endometrial cancer. If immunotherapy use can mitigate disparities in outcomes for patients with endometrial cancer, as these preliminary, hypothesis-generating data suggest, ensuring equitable access to early diagnosis, molecular testing, and emerging therapies is vital, especially in a rapidly changing treatment environment. Future studies incorporating socioeconomic, clinical, and healthcare access measures are needed to better characterize factors underlying these disparities.

## Figures and Tables

**Table 1:** Demographic and socioeconomic characteristics of patients receiving immunotherapy as front-line therapy by race and ethnicity.

	Asian	Hispanic	Non-Hispanic Black	Non-Hispanic White	Total	*p*-Value
	*n* = 182 (4%)	*n* = 292 (7%)	*n* = 912 (21%)	*n* = 2936 (68%)	*n* = 4322	
Age (Years)						<0.001
18–49	14 (8)	59 (20)	30 (3)	164 (6)	267	
50–69	126 (69)	157 (54)	617 (68)	1662 (57)	2562	
70+	42 (23)	76 (26)	265 (29)	1110 (38)	1493	
Year of diagnosis						0.772
2019	23 (13)	49 (17)	142 (16)	470 (16)	684	
2020	36 (20)	72 (25)	216 (24)	659 (22)	983	
2021	59 (32)	75 (26)	258 (28)	811 (28)	1203	
2022	64 (35)	96 (33)	296 (32)	996 (34)	1452	
Insurance						<0.001
Private	90 (49)	106 (36)	316 (35)	1032 (35)	1544	
Medicaid	22 (12)	58 (20)	105 (12)	169 (6)	354	
Medicare	60 (33)	103 (35)	449 (49)	1670 (57)	2282	
Unknown/missing/uninsured	10 (5)	25 (9)	42 (5)	65 (2)	142	
Neighborhood education attainment (% of zip code with high school degree)						<0.001
Below median	51 (28)	173 (59)	513 (56)	877 (30)	1614	
Above median	114 (63)	72 (25)	262 (29)	1574 (54)	2022	
Missing/unknown	17 (9)	47 (16)	137 (15)	485 (17)	686	
Neighborhood household income (% of zip code with median income of $50,353)						<0.001
Below median	24 (13)	120 (41)	434 (48)	806 (27)	1384	
Above median	141 (78)	125 (43)	341 (37)	1645 (56)	2252	
Missing/unknown	17 (9)	47 (16)	137 (15)	485 (17)	686	
Charlson Comorbidity Score						0.009
None	142 (78)	214 (73)	639 (70)	2211 (75)	3206	
1 or more	40 (22)	78 (27)	273 (30)	725 (25)	1116	
Facility type **						<0.001
Academic/research	71 (40)	156 (58)	485 (53)	1215 (42)	1927	
Integrated network	37 (21)	43 (16)	148 (16)	571 (20)	799	
Community (comprehensive community and community)	70 (39)	72 (27)	274 (30)	1102 (38)	1518	
Distance from treatment facility **						<0.001
Less than 50 miles	155 (94)	223 (90)	730 (93)	2053 (83)	3161	
More than 50 miles	10 (6)	24 (10)	51 (7)	422 (17)	507	
Geographical region **						<0.001
Northeast	45 (25)	50 (18)	240 (26)	704 (24)	1039	
Midwest	13 (7)	23 (8)	161 (18)	840 (29)	1037	
South	37 (21)	109 (40)	454 (50)	868 (30)	1468	
West	83 (47)	89 (33)	52 (6)	476 (16)	700	
Urban Density **						<0.001
Metropolitan	*	275 (95)	817 (93)	2328 (80)	*	
Non-metropolitan (urban and rural)	*	14 (5)	62 (7)	553 (20)	*	

* Cells with *n* < 10 are intentionally repressed in accordance with NCDB data use agreement to protect patient confidentiality. Totals suppressed where necessary. ** Percentages are calculated among patients with non-missing data due to NCDB data use agreement for small cell suppression in missing cells.

**Table 2:** Clinical characteristics of patients receiving immunotherapy as front-line therapy by race/ethnicity.

	Asian	Hispanic	NHB	NHW	Total	*p*-Value
	*n* = 182 (4%)	*n* = 292 (7%)	*n* = 912 (21%)	*n* = 2936 (68%)	*n* = 4322	
Stage **						0.052
Early (I–II)	20 (11)	26 (9)	79 (9)	340 (12)	465	
Advanced (III–IV)	158 (89)	249 (91)	785 (91)	2419 (88)	3611	
Histology						<0.001
Endometrioid	59 (32)	101 (35)	144 (16)	954 (32)	1258	
Serous	68 (38)	101 (35)	467 (51)	1071 (36)	1707	
Carcinosarcoma	12 (7)	16 (5)	95 (10)	188 (6)	311	
Other	43 (24)	74 (25)	206 (23)	723 (25)	1046	

** Percentages are calculated among patients with non-missing data due to NCDB data use agreement for small cell suppression in missing cells.

**Table 3:** Factors associated with overall survival amongst patients with endometrial cancer who received immunotherapy as front-line therapy.

	HR	95% CI
Age	1.01	1.00–1.03
Race		
Non-Hispanic White	Reference	
Non-Hispanic Black	1.03	0.86–1.23
Hispanic	1.37	1.05–1.80
Asian	1.12	0.78–1.61
Insurance		
Private	Reference	
Uninsured	0.92	0.55–1.53
Medicaid	1.00	0.76–1.31
Medicare	1.00	0.83–1.19
Neighborhood education attainment (% zip code with high school degree)		
Below US median	Reference	
Above US median	0.85	0.72–1.00
Neighborhood Income (% of zip code with median income of $50,353)		
Below median	Reference	
Above median	1.03	0.87–1.22
Charlson comorbidities		
None	Reference	
More than one	1.1	0.95–1.28
Distance from treatment facility		
Less than 50 miles	Reference	
More than 50 miles	0.91	0.73–1.13
Facility type		
Academic	Reference	
Integrated network	1.10	0.91–1.32
Community hospital	1.02	0.87–1.19
Geographic region		
Northeast	Reference	
Midwest	1.30	1.07–1.59
South	1.07	0.89–1.29
West	1.13	0.90–1.41
Urban density		
Metropolitan	Reference	
Non-metropolitan	0.90	0.72–1.13
Stage		
Early (I–II)	Reference	
Advanced (III–IV)	3.10	2.27–4.26
Histology		
Endometrioid	Reference	
Serous	1.27	1.05–1.53
Carcinosarcoma	2.32	1.79–3.00
Other	1.67	1.36–2.03

## Data Availability

All data is publicly available on the National Cancer Database website https://www.facs.org/quality-programs/cancer-programs/national-cancer-database/ (accessed on 22 October 2024).

## Abbreviations

The following abbreviations are used in this manuscript:

dMMR	Mismatch Repair Deficient
FDA	Food and Drug Administration
FIGO	Federation of Gynecology and Obstetrics
MSI-H	Microsatellite Instability High
NCDB	National Cancer Database
NHB	Non-Hispanic Black
NHW	Non-Hispanic White
US	United States
